# Open questions in marine mammal sensory research

**DOI:** 10.1242/bio.059904

**Published:** 2023-03-21

**Authors:** Steffen De Vreese, Kenneth Sørensen, Kristy Biolsi, Jeffry I. Fasick, Joy S. Reidenberg, Frederike D. Hanke

**Affiliations:** ^1^ Laboratory of Applied Bioacoustics, Technical University of Catalonia (BarcelonaTech), 08800 Vilanova i la Geltrù, Spain; ^2^ University of Rostock, Institute for Biosciences, Neuroethology, Albert-Einstein-Str. 3, 18059 Rostock, Germany; ^3^ Department of Psychology, St. Francis College, Brooklyn NY 11201, USA; ^4^ Center for the Study of Pinniped Ecology and Cognition (C-SPEC), Brooklyn Heights, USA; ^5^ Department of Biological Sciences, University of Tampa, Tampa, FL 33606, USA; ^6^ Center for Anatomy and Functional Morphology, Icahn School of Medicine at Mount Sinai, 1 Gustave L. Levy Place, Mail Box 1007, New York, NY 10029-6574, USA

**Keywords:** Semi-aquatic lifestyle, Senses, Sensory ecology

## Abstract

Although much research has focused on marine mammal sensory systems over the last several decades, we still lack basic knowledge for many of the species within this diverse group of animals. Our conference workshop allowed all participants to present recent developments in the field and culminated in discussions on current knowledge gaps. This report summarizes open questions regarding marine mammal sensory ecology and will hopefully serve as a platform for future research.

## Teaser

**Summary:** The report summarizes future areas of marine mammal sensory research including aerial sensing in fully aquatic species, size-related questions in large species, seemingly vestigial structures, and concerted action of senses.

## Introduction

Marine mammals, including cetaceans, pinnipeds, sirenians, sea otters, and polar bears (Marine Mammal Commission, 1972), are secondarily adapted to the aquatic environment. Over the course of evolution, both the anatomy and physiology of the respective terrestrial ancestors were extensively modified to adapt to the requirements of either a semi- or fully aquatic lifestyle (Berta et al., 2005). These secondary adaptations also resulted in specific modifications of the sensory systems, which act as the interfaces between an organism and its environment. They provide information about the external world necessary for critical tasks such as foraging, orientation, navigation, threat detection, conspecific recognition, and reproduction. After decades of research on marine mammal sensory systems, our understanding of the behaviors mediated by the sensory systems and the sensory organs has generally increased albeit mostly in only a few species. Nevertheless, numerous key questions remain unresolved.

The workshop ‘Marine Mammal Sensory Systems: Recent Advances and Emerging Technologies’ was held as a hybrid workshop prior to the Biennial Conference on the Biology of Marine Mammals of the Society for Marine Mammalogy in Palm Beach, USA, in July 2022. It focused on recent developments in marine mammal sensory research, including new methodologies that have led to new insights into how these sensory systems work and how they interact with each other. It provided a platform for early career scientists to present their research in short oral presentations and fostered discussions with senior experts in the field, to advance individual projects as well as the field of marine mammal sensory systems in general. The format of the workshop resulted in an inspiring atmosphere that gave prominence to four major directions on which this report is focused.

## Sensing in different media

The adaptation of the senses to the aquatic environment involved several specializations to detect stimuli in this medium. Some marine mammals such as pinnipeds, sea otters, and polar bears, have a semi-aquatic or amphibious lifestyle, whereas cetaceans and sirenians are fully aquatic. While previous research on sensory systems of semi-aquatic marine mammals has considered challenges associated with amphibious sensing (see e.g. Reichmuth et al., 2013), research on fully aquatic marine mammals has largely excluded aerial sensing (but see e.g. Herman et al., 1975). Nevertheless, when fully aquatic marine mammals surface (e.g. for breathing, spyhopping, or performing aerial maneuvers), they likely use their senses in air. However, as aerial sensing has not been a focus of marine mammal sensory research with respect to the fully aquatic species to date, it is largely unknown to what extent these sensory modalities contribute to aerial behaviors. Amphibious sensing would provide an entirely new perspective on sensory systems in fully aquatic marine mammals and may shed new light on for example smell (olfaction) and taste (gustation) functions, particularly in cetaceans. Olfaction and gustation (i.e. chemoreception in general), are largely understudied and many questions as basic as ‘What (if anything) can cetaceans smell or taste?’ are still unclear (Bouchard et al., 2019; Bouchard et al., 2022; for review see Kremers et al., 2016). The proposed perspective on marine mammal sensing, including aquatic and aerial sensing, will require examining adaptations that allow for amphibious sensing in all marine mammal species.

When considering amphibious sensing in marine mammals, researchers should also rethink the sensory cues that are available as well as those being used in numerous behavioral contexts. This aspect has large implications concerning the sizes of the sensory windows, the range of sensitivity of the senses, to be considered. These sensory windows might be broader than currently thought or specific aerial and aquatic sensory windows might exist. Sensory windows are generally difficult to assess, and some marine mammals (e.g. echolocating odontocetes) are known to passively and actively adjust their sensory windows either to protect sensory organs from excessive stimulation or optimize gain control during daily activities (see for example Harley et al., 2022; Kloepper et al., 2014). The adaptability of sensory windows and their relation to the sensory cues available is an aspect that requires further study.

Valuable insight into sensory cues available in air and underwater is expected to be gained from deploying state-of-the-art biologgers such as animal-borne cameras, hydrophones, and environmental data loggers on marine mammals (e.g. Tyack et al., 2006). The deployment of such data loggers allows the recording of numerous parameters (e.g. ambient light, salinity, depth/pressure, sound, temperature, velocity, acceleration, orientation), while the animal is cruising in its habitat as a ‘living environmental sensor’ (Couzin and Heins, 2022; Ropert-Coudert and Wilson, 2005). Thus, developments in the field of data-/biologging, complemented by ongoing exploration and mapping of the ocean environment, and also animal-borne cameras will broaden our understanding of sensory cues available to marine mammals in their environment. From this, we will gain insight into the life of these organisms which is often enigmatic and inaccessible to humans. Information on the availability of sensory cues provided by biologgers will in return also allow exploring specific questions about the sensory systems involved in perceiving these cues and the corresponding sensory windows to be considered.

## Size-related physiological adaptations of the sensory systems and central processing

Among marine mammals, differences in anatomical scale are immense. In this group of animals, we can find animals as small as a vaquita (*Phocoena sinus*), a female Galapagos fur seal (*Arctocephalus galapagoensis*), or a sea otter (*Enhydra lutris*), while at the same time, it encompasses among the largest animals living on earth, such as the blue whale (*Balaenoptera musculus*, the largest mysticete) and the sperm whale (*Physeter macrocephalus*, the largest odontocete). The physiology of the sensory systems, from peripheral nerves and sensory receptors to the central nervous system, is likely to show specific specializations for size, an aspect that needs to be systematically examined in marine mammals in future studies. In somatosensation, for example, the distance a signal must travel from peripheral sensory receptors to the brain is long in large animals such as in mysticetes, for which somatosensation is especially under-examined (but see Eldridge et al., 2022). These large whales likely benefit from axons with high transduction velocities to avoid delays in sensory perception. Moreover, the amount of somatosensory information perceived over the large body surface is expected to exceed the amount of information being processed by small animals. Unsurprisingly, we find large parts of, for example, the sirenian brain, to be devoted to somatosensory processing, including the processing of the input obtained by their vibrissae (review in Bauer et al., 2018; Sarko et al., 2007; Sarko and Reep, 2007, 2022).

The physiological costs and benefits might also be balanced differently in large animals compared with smaller animals. For example, the largest eyes in the animal kingdom are found in marine mammals, although the eyes are smaller than expected when considering their body mass and allometric relations found in other animals (Harvey, 2019). A larger eye might be advantageous for vision, but further enlarging a big eye by an absolute amount does not result in the same improvement as enlarging a small eye by the same amount according to the law of diminishing returns (Nilson et al., 2012). Most likely, the improvement brought about by increasing eye size needs to be balanced with the larger energetic costs for maintenance arising from the specific increase in size.

It needs to be noted that the large body sizes, as well as sensory organ sizes, pose challenges to the experimental approaches and technological equipment to study them. As mysticetes are large, it is impossible to maintain them in captivity to perform robust behavioral studies. In this context, it is not surprising that our knowledge about sensory behavior is limited to smaller marine mammals such as harbor seals (*Phoca vitulina*), California sea lions (*Zalophus californianus*), and bottlenose dolphins (*Tursiops truncatus*; for review see Hanke and Reichmuth, 2022; Hanke et al., 2021; Hanke and Erdsack, 2015). Our current limited knowledge of the sensory systems in mysticetes is mainly deduced from opportunistic anatomical sampling or behavioral observations of free-ranging animals. Therefore, researchers are challenged to find novel approaches to get a deeper insight into mysticetes' sensory systems, including new state-of-the-art technologies.

## Seemingly vestigial structures might have functions

When comparing sensory systems across marine mammals, some anatomical structures that were previously classified as vestigial might serve a biologically important function. To give an example, most cetaceans are born with vibrissae on their rostrum. While a vibrissal hair can be observed within these crypts before and shortly after birth, the hair is lost after a few weeks, at least in most odontocete species (Gerussi et al., 2021; Ling, 1977; Mynett et al., 2022). While the vibrissae in neonates most likely serve a still unidentified mechanosensory function, the remaining crypts were described as ‘vestigial’ (Ling, 1977; Yablokov and Klevezal, 1969), but new research reveals that these crypts can function as electroreceptors (Hüttner, 2022; Hüttner et al., 2022). Similarly, the remnants of the outer ear canal in all cetaceans studied to date seem to have transformed the structure from a sound conductor into a mechanosensory organ that could act as a barometer in these diving animals (De Vreese et al., 2014, 2020). Considering these two examples, when studying marine mammal sensory systems, researchers should not overlook seemingly vestigial anatomical structures.

Moreover, as sensory systems and modalities can overlap, there may not be clear boundaries in sensory perception. For example, a sea quake can be ‘heard’ but equally ‘felt’. This questions the value of our current classification of marine mammal sensory perception/senses purely based on anatomy. The field of marine mammal sensory research might benefit from freeing from the classic concepts of defining senses by anatomy (compare with the phenomenological approach described by, for example, Merleau-Ponty, 1966).

## Concerted action of sensory systems

Current research on marine mammal senses typically focuses on a single sense (but see Bruck et al., 2022; Charrier et al., 2022; Harley et al., 2003; Pack and Herman, 1995). However, during everyday activities, the different senses work together to create a multimodal representation of the environment highlighting the need to investigate sensory integration in marine mammals and beyond (Johnsen, 2017). Previous work on terrestrial mammals has shown for example that the integrated response to a combination of weak unisensory signals is enhanced when compared to the response elicited by the unisensory signal (Stein and Meredith, 1993; Stein et al., 1989). The first cross-/multimodal approaches adopted in marine mammal research (for reviews see Bruck and Pack, 2022; Charrier et al., 2022) already revealed interesting insight.

While sensory integration can be approached through behavioral experiments, these aspects could also be investigated directly by looking at processes within the brain. Functional brain measurements in conscious marine mammals could reveal where and how information from different senses is being integrated (see first approaches/ideas in McKnight et al., 2021; Ruesch et al., 2022). For example, the superior colliculi (a brain structure highly responsible for integrating somatosensory, visual, and auditory information to initiate motor commands in humans) have been heavily studied as a potential model system for multisensory integration in animals (Stanford et al., 2005; Wallace and Stein, 1996). Brain imaging techniques combined with postmortem assessments of central sensory processing structures (e.g. Cook et al., 2018; Orekhova et al., 2022) will most likely contribute greatly to a comprehensive understanding of how sensory information of multiple modalities is integrated and used to perform complex behaviors. Such an approach could also help to discern adaptations for sensory perception in the three-dimensional underwater environment allowing movements with all degrees of freedom (Cook and Berns, 2022).

## Conclusions

The workshop helped define key topics for future marine mammal sensory research, including further investigations of understudied sensory organs, integrated sensory capabilities and functions in different media, the effect of size on sensory system performance, and the utilization of new technologies to study sensory abilities. The field will benefit from even more interdisciplinary collaborations, providing platforms for sharing resources, expertise, data, or even access to samples. A greater understanding of sensory science is, beyond fundamental science, the key to correctly identifying and assessing potential risks to marine mammal populations, and developing long-term management strategies that protect and conserve marine mammals and their habitats.
